# Exploring Australian child and youth mental health occupational therapists’ perception and utilisation of creative activities: A survey snapshot

**DOI:** 10.1177/03080226251412760

**Published:** 2026-02-26

**Authors:** Hannah Forbes, Adam Lo, Michael Steele, Rosamund Harrington

**Affiliations:** 1 School of Allied Health, Australian Catholic University, Banyo, QLD, Australia; 2 Metro South Addiction and Mental Health Services, Queensland Government, Logan Central, QLD, Australia

**Keywords:** Occupational therapy, creativity, mental health, child health, professional practice

## Abstract

**Introduction:**

Creative activities have traditionally been an important therapeutic medium in occupational therapy; however, research suggests a decline in their perceived efficacy, utilisation, and teaching. This study explored Australian child and youth mental health (CYMH) occupational therapists’ perceptions and use of creative activities.

**Method:**

A 31-item web-based survey, comprising quantitative and qualitative questions, was disseminated via convenience sampling to collect data from Australian CYMH occupational therapists. Descriptive statistics and non-parametric Mann–Whitney U tests were used for quantitative data, with inductive content analysis used for qualitative data.

**Results:**

Analysis of 33 completed surveys revealed creative activities were perceived as a highly efficacious therapeutic medium (78%), utilised most frequently multiple times a week (66%). All respondents utilised creative activities, although 64% were not content with their current use. Although 67% rated confidence using creative activities highly, 97% identified additional training needs to feel more confident. Three main categories central to use of creative activities arose: ‘creative activities as a therapeutic medium’, ‘legitimacy of creative activities within occupational therapy’ and ‘initial and ongoing professional development’.

**Conclusion:**

Australian CYMH occupational therapists perceived and utilised creative activities positively. Lack of discipline-specific evidence and unmet training needs impacted confidence in their use.

## Introduction

Over the last decade, there has been growing international research exploring the relationship between arts and health (Fancourt and Finn, 2019). A recently published nationally representative sample of the Australian population aged 15–65+ (*n* = 9396), found an overwhelming 97% of Australians engage with the arts, 44% engage in creative participation, and 56% perceive the arts have a ‘big’ or ‘very big’ impact on sense of wellbeing and happiness (Creative Australia, 2023). Although young Australians (15–24) are the most likely of all the age groups to participate and benefit from creative engagement (Creative Australia, 2023), youth mental health remains a pressing public health issue. According to the second national survey of the mental health and wellbeing of Australian children and adolescents, approximately one-fifth of adolescents (aged 11–17) reported experiencing high to very high levels of psychological distress over the preceding 4 weeks (Lawrence et al., 2015). Moreover, despite coordinated prevention initiatives, suicide remains the leading cause of death among 15- to 24-year-olds (Australian Institute of Health and Welfare, 2025).

Fortunately, there is considerable inter-disciplinary evidence for the effectiveness of creative occupation and activity-based interventions in promoting mental health, preventing psychological distress and intervening in mental health concerns among children and youth (Arbesman et al., 2013; Cahill et al., 2020; Fancourt and Finn, 2019). Occupation-based interventions, which use engagement in meaningful and fulfilling occupation as the therapeutic agent of change, may be particularly relevant in this context (Ashby et al., 2013). The occupational therapy profession has a long history of utilising creative therapies in mental health settings (Hansen et al., 2021, 2024); however, literature remains lacking (Johnson et al., 2022), particularly discipline-specific evidence of creative activities in child and youth mental health (CYMH; Ashby et al., 2013).

In lieu of discipline-specific evidence, occupational therapists can integrate evidence from multiple disciplines to strengthen evidence of the benefits of creative occupations and interventions in CYMH (Arbesman et al., 2013). Recent systematic and scoping reviews have found moderate-to-strong evidence for benefits of arts participation on social interaction skills for children with autism (Arbesman et al., 2013; Bernier et al., 2022) and low evidence for arts interventions reducing mental-health-related stigma among youth (Gaiha et al., 2021). Similar reviews have concurred positive benefits of creative activities for children and adolescents’ behaviour, self-esteem, self-confidence and self-worth (Bungay and Vella-Burrows, 2013; Fancourt and Finn, 2019), as well as emotional regulation, cognitive flexibility and social connectedness influenced by engagement in creative activities across the lifespan (Jean-Berluche, 2024). However, reported methodological weaknesses (Bungay and Vella-Burrows, 2013), small sample sizes (Jean-Berluche, 2024) and lack of comparability across studies (Bernier et al., 2022) are limitations of the existing evidence base.

Creative activities have long been recognised as a valuable therapeutic medium within occupational therapy, particularly in the field of mental health (Levine, 1987; Lloyd and Papas, 1999). Creative activities are described as an individual’s involvement in originative, productive and purposeful activity, using imagination and creative skills (Cynkin and Robinson, 1990). These activities have included, but are not limited to, arts, crafts, pottery/ceramics, music, drama and photography (Griffiths and Corr, 2007; Müllersdorf and Ivarsson, 2012). Such creative activities align with the philosophical underpinnings of occupational therapy, which focus on the importance of engagement in meaningful activities to enhance overall health, wellbeing and quality of life (Polatajko et al., 2013). In addition, the use of creative occupations in therapy has been associated with enabling expression and fostering adjustment to life’s challenges (Thompson and Blair, 1998). The Vivaio model (MOVI), a relational model of occupational therapy, highlights the value of ‘doing’ and spontaneous interactions during free play and creative activities in fostering transformative therapeutic relationships over time (Cunningham-Piergrossi and de Sena-Gibertoni, 2013).

Central to the value of ‘doing’ and engagement in occupations embedded in occupational therapy philosophy, creative activities were once incorporated as a key element of occupational therapy education in Australia and internationally (Coppola, et al., 2017; Farnworth and Kennedy-Jones, 2017; Schmid, 2004). However, research highlights a notable decline in their support and inclusion in occupational therapy training (Bathje, 2012; Lloyd and Papas, 1999), with some authors suggesting creative activities are admonished in comparison to more evidence-based interventions (Kreider et al., 2014). A growth in arts and crafts participation at a societal level, and a re-focus on occupation-based interventions, has encouraged renewed interest in the perception and utilisation of creative activities in occupational therapy practice (Bathje, 2012). Cross-sectional surveys of occupational therapists in Europe and the United Kingdom (Griffiths and Corr, 2007; Müllersdorf and Ivarsson, 2012) have demonstrated frequent use of creative activities across practice settings. Specifically, Griffiths and Corr (2007) found 82% of surveyed mental health occupational therapists in the United Kingdom used creative activities as an intervention, whilst adult mental health practitioners positively supported the use of creative activities in enhancing therapeutic relationships, self-expression, identity, recovery, and client well-being (Lloyd et al., 2007; Müllersdorf and Ivarsson, 2016).

Occupational therapists in CYMH work in multidisciplinary teams to support children and youth with complex mental health needs under the age of 18 (Children’s Health Queensland, 2025). Anecdotally, occupational therapists working in CYMH use creative activities to achieve similar benefits to those outlined above; however, a lack of discipline-specific evidence to inform practice is a key barrier to service provision (Hardaker et al., 2011). In a review of creative arts occupations in therapeutic practice, Perruzza and Kinsella (2010) found only 8 out of 23 studies specifically examined creative activity use by occupational therapists and only one targeted children as participants. Considering the dearth of evidence in CYMH occupational therapy practice, especially in Australia, understanding of the perceived efficacy and utilisation of creative activities is unclear. This study aimed to respond to this existing gap, through addressing the following research questions:

1. What is the current utilisation of creative activities by Australian CYMH occupational therapists?
2. What are Australian CYMH occupational therapists’ perceptions of the purpose and efficacy of using creative activities with children and youth?
3. What factors influence Australian CYMH occupational therapists’ utilisation and perceptions of creative activities?

## Methodology

A cross-sectional survey study was completed to provide a complementary discussion using quantitative and qualitative data, to enrich understanding of unique perspectives and lived experiences (Creswell and Plano Clark, 2011). A survey design was implemented to generate a breadth of insights into Australian CYMH occupational therapists’ perceptions and utilisation of creative activities. Ethics approval was granted by the Metro South Human Research Ethics Committee and the Australian Catholic University Human Research Ethics Committee (HREC/16/QPAH/816). Unique identifier codes and pseudonyms were assigned to each participant during analysis and publication, to ensure anonymity and track responses for data analysis.

### Sampling and recruitment

Australian CYMH occupational therapists were recruited using convenience sampling and snowball recruitment techniques via a web-based survey link including study information, which was disseminated on two occasions via the Occupational Therapy Australia eNewsletter and CYMH special interest group. Therapists were included in the study if they: currently or had within the past 5 years practised in CYMH in Australia; and either did or did not use creative activities. Therapists with higher education/degrees in music or art therapy were excluded, as it was viewed they would have strong opinions about the value of creative activities as a therapeutic medium which may or may not be related to the occupational therapy scope of practice and may skew results.

### Data collection procedures

A web-based survey design was chosen, as it is safe, confidential and time-efficient for participants (Creswell and Plano Clark, 2011) and appropriate for capturing therapists’ perspectives from across Australia in a short time frame. The web-based survey developed by the research team, informed by a review of related occupational therapy and allied health research evidence, is available (refer to Supplemental Material). The survey was piloted by five experienced mental health occupational therapists who provided feedback on the design. The survey tool was then modified based on feedback provided, to enhance readability, accessibility and relevance of the tool. All pilot data was removed prior to activation of the survey link using Qualtrics software (https://www.qualtrics.com).

The final 31-item survey included a mixture of questions to allow fixed, open-ended and ranking responses, spread across two sections and remained active for responses from participants for a period of 8 weeks. Section 1 comprised 10 fixed response questions capturing consent and demographic information, followed by 14 questions of either fixed, open-ended or ranking responses to questions examining therapists’ aims/outcomes, utilisation of and training and confidence in creative activities. Section 2 included seven questions allowing fixed or open-ended responses, exploring education/training and recommendations for creative activity use in CYMH occupational therapy practice. The open-ended survey questions asked therapists about their expected outcomes when using creative activities, barriers and enablers to creative activity use, recommendations for training in creative activities for occupational therapy students and suggestions to improve/promote creative activities in Australian occupational therapy practice.

### Data analysis

Quantitative data captured and stored in Qualtrics was entered into IBM SPSS Statistics Version 22 (IBM Corp., 2014). For demographic and content response variables frequencies and percentages were calculated. Non-parametric Mann–Whitney U tests and correlations were used with statistical significance set to *p*-values of less than 0.05. Correlations between several content response variables were undertaken between age, years of practice, workplace setting, use of creative activities, perceived confidence, perceived expertise, contentment with extent of use of creative activities, types of training and types of creative activities used. Inductive content analysis of qualitative survey data was undertaken (Kyngäs, 2020), following processes outlined by Elo and Kyngäs (2008). In the preparation phase, qualitative survey data for each open-ended question was extracted, collated and recorded in tabulated form in an Excel file and read multiple times. All members of the research team independently completed open coding of survey responses, and subsequently met repeatedly to discuss and compare coding, with lists of categories grouped under higher order categories. The first author then developed a description for each category, and all research team members reviewed the list of categories and sub-categories to ensure an accurate depiction of experiences and perspectives shared by survey participants. In addition to peer coding, an audit trail, reflexive journaling and peer debriefing were undertaken to document analytic decisions and reduce bias (Nowell et al., 2017). Credibility and transferability were further supported through triangulation of survey responses, team discussions, thick description of participant contexts and member checking of survey data with three experienced clinicians (Ahmed, 2024).

## Results

### Quantitative findings

A total of 36 surveys were returned, with three later excluded from analysis as two respondents did not have recency of practice in Australia, and the third had higher education in music/art therapy. Reported results include responses from 33 therapists; however, some questions only 31 therapists responded, and this is reported accordingly. Detailed overview of participant demographics are presented in Table 1, with a brief outline provided below.

**Table 1.** Participant demographic information.

Characteristics	*n* (%)
Gender	
Female	29 (88)
Male	4 (12)
Age (years)	
21–40	25 (76)
41–60	8 (24)
Location	
Queensland	19 (58)
New South Wales	6 (18)
South Australia	4 (12)
Victoria	3 (9)
Western Australia	1 (3)
Practical experience	
Less than 5 years	14 (42)
5 or more years	19 (58)
Level of qualification	
Bachelor’s degree	21 (64)
Greater than bachelor’s degree	12 (36)
Level of specialisation in mental health	
Entry grade occupational therapist	5 (14)
Experienced occupational therapist	10 (31)
Senior occupational therapist	15 (46)
Head occupational therapist	2 (6)
Other	1 (3)
Employment status	
Full-time	19 (59)
Part-time	13 (38)
Other	1 (3)
Primary place of employment	
Public hospital	13 (40)
State government	6 (18)
Private practice	5 (15)
Community service provider	5 (15)
Private hospital	2 (6)
Other	2 (6)
Primary practice setting	
Community	22 (67)
Acute hospital unit	5 (15)
Other	6 (18)

#### Characteristics of study participants

Of the 33 participants, most were women (88%), and almost half were senior occupational therapists (46%). Five of seven Australian states were represented in the data, with a large proportion of respondents residing in Queensland (58%). Just under half (40%) reported public hospitals as their primary place of employment, with approximately two-thirds (67%) identifying community practice as their primary practice setting.

#### Perceived efficacy of creative activities

Participants rated how effective they perceived creative activities as an intervention on a Likert scale from 0 (not at all effective) to 10 (extremely effective). All therapists rated creative activities above 5, and 78% rated them highly effective (⩾7/10). Almost all therapists (90%) who received creative activity education/training in undergraduate occupational therapy or formal workplace training in creative activities perceived them a highly effective therapeutic medium. Most therapists (70%) who had not received undergraduate occupational therapy or formal workplace training perceived creative activities to be a highly effective therapeutic medium.

#### Utilisation of creative activities

All therapists (*n* = 33) identified using creative activities in practice: 30% of therapists monthly, 42% multiple times/week, and 24% daily/multiple times daily. Therapists with formal workplace training were more likely to use creative activities multiple times/week or more, compared to those without (82% and 55%). Therapists were most likely to use creative activities with children aged 13–15 (85%) compared to those aged 0–4 (36%). Self-esteem (97%), interpersonal skills (82%) and social skills (79%) were the most common occupational challenges experienced by children seen. Creative activities were used as a therapeutic intervention for children with anxiety (100%), depression (87%) and attachment disorders (87%), most frequently aiming to promote therapeutic relationships (45%) and self-expression (29%; *n* = 31). Creative activities used as interventions are reported in Table 2.

**Table 2.** Key quantitative findings from survey data analysis.

Descriptors	Answering options	Frequency (*n* = 33)^a^ (*n* = 31)	Percentages (*n* = 100)
Age group most likely to use creative activities as an intervention	0–4	12	36
	5–8	22	67
	9–12	23	70
	13–15	28	85
	16–18	23	70
Conditions therapists most likely to use creative activities for children with anxiety	Depression	27	87
	Anxiety	31	100
	Mood disorders	26	84
	Attachment disorders	27	87
	Social phobia	21	68
	Obsessive compulsive disorder	15	48
	Psychotic disorders	15	48
	Other	7	23
Areas of difficulty most reported by children accessing CYMH services	Self-esteem	32	97
	Motivation	19	58
	Concentration	25	76
	Social skills	26	79
	Self-management	22	67
	Interpersonal skills	27	82
	Other	5	15
Creative activities most commonly used as an intervention^a^	Painting	22	71
	Artwork	27	87
	Drawing/colouring	30	97
	Collage	20	65
	Paper-craft	22	71
	Printing	5	16
	Photography	9	29
	Calligraphy	1	3
	Sewing	2	6
	Patchwork	0	0
	Knitting	3	10
	Pottery	3	10
	Sculpture	10	32
	Drama	9	29
	Creative writing	13	42
	Dance	9	29
	Music	17	55
	Other	5	16
Most important aim/outcome using creative activities in CYMH^a^	Self-esteem	3	9
	Self-confidence	1	3
	Social engagement	0	0
	Therapeutic relationships	14	42
	Sense of achievement	1	3
	Enhancing task performance	2	6
	Self-expression	9	27
	Assessment	1	3

CYMH: child and youth mental health.

a Results reported in these sections feature responses from 31 participants.

#### Perceived confidence and expertise in creative activities

Approximately two-thirds of the therapists (67%) rated their confidence using creative activities highly (7–10), 24% felt moderately confident and 9% did not feel confident (*n* = 33). Therapists who received undergraduate occupational therapy or formal workplace training in creative activities reported significantly higher confidence than those who had not (*p* *=* 0.017 and *p* = 0.035, respectively). Almost half (49%) of therapists rated their level of expertise highly (*n* = 33). Therapists who had received formal workplace training were more likely to rate their expertise in creative activities higher than those who had not (*p* *=* 0.020). More than half of the therapists (64%) expressed discontent with the extent to which they currently used creative activities and almost all (97%) felt they would be more confident if provided with additional training (*n* = 33). Further, a strong positive correlation between therapists’ confidence and perceived level of expertise using creative activities was identified (*r* = 0.816, *p* < 0.001). Therapists who rated their level of expertise highly, were more likely to be content with the extent of their use of creative activities (*p* *=* 0.013) and have higher levels of confidence (*p* = 0.004). Significant relationships were not found between therapists’ rated confidence or level of expertise using creative activities and their age, experience, qualifications or completion of informal or formal training outside of their workplace.

### Qualitative findings

Analysis of qualitative survey responses identified three main categories central to the use of creative activities: ‘creative activities as a therapeutic medium’, ‘legitimacy of creative activities within occupational therapy’ and ‘initial and ongoing professional development’. Visual depiction of the 3 main categories, 7 generic categories and 22 sub-categories can be seen in Table 3. Pseudonyms have been used throughout to give voices to survey participants responses.

**Table 3.** Main categories arising from qualitative data analysis.

Main category	Generic category	Sub-categories
Creative activities as a therapeutic medium	Aims and outcomes (*n* = 33) Therapeutic approach (*n* = 13) Barriers and enablers (*n* = 30)	Sense of self-control/self-regulation (*n* = 10) Self-expression/communication (*n* = 14) Self-identity (*n* = 9) Sense of mastery/achievement (*n* = 9) Engagement and participation in meaningful activity (*n* = 15) MOVI theoretical framework (*n* = 3) Therapeutic approach/process (*n* = 10) Fluidity and flexibility (*n* = 5) Valid assessment/observation method (*n* = 8) Creative activities as a means and ends (*n* = 6) Comfort, clinical utility and workplace supports (*n* = 30) Funding and resource constraints (*n* = 21)
Legitimacy of creative activities within occupational therapy	Evidence-informed practice (*n* = 20) Ownership by the profession (*n* = 10)	Lack of international occupational therapy research (*n* = 11) Limited Australian studies in occupational therapy (*n* = 9) Devaluation of occupation-based interventions (*n* = 10) Inter-professional and multidisciplinary collaboration (*n* = 13)
Initial and ongoing professional development	Organisational/corporate governance (*n* = 21) Undergraduate education and training (*n* = 17)	Formal vs informal training and education (*n* = 15) Experiential learning (*n* = 13) Parent education (*n* = 6) Knowledge translation (*n* = 11) Undergraduate training (*n* = 9) Further education/higher training needs (*n* = 8)

#### Creative activities as a therapeutic medium

##### Aims and outcomes

Therapists reported a diverse range of expected outcomes of using creative activities, including engagement/participation in meaningful activity (*n* = 15), enhanced self-expression/communication (*n* = 14), sense of control/self-regulation (*n* = 10), self-identity (*n* = 9), and mastery/achievement (*n* = 9). Aims and outcomes expressed by one therapist included:

Alternative way to help client create meaning for their experience, to create a visual representation not relying on language-based intervention, and identify strengths, likes, and dislikes to help foster a sense of identity. (Leanne, S19)

Nine therapists perceived creative activities as a powerful language for self-expression, reported to provide ‘clarity and insight for the young person. . .an alternative to verbalising experiences’ (Mary, S9) and ‘increase confidence through engagement in creative and meaningful occupation’ (Anna, S10). Creative activities were perceived favourably for their unique ability to build connections and communicate through ‘activity-based expression. . .allowing emotional and physiological safety, and self-regulation, thinking and mental organisation’ (Sally, S12). Furthermore, creative activities were viewed as beneficial in allowing for mastery including ‘enhanced motor planning; utilisation of fine motor skills; challenge sensory processing issues; improve social engagement, turn taking, communication, sharing; and improved sense of achievement and confidence’ (Ashley, S18).

##### Therapeutic approach/process

Therapists (*n* = 10) highlighted the value of creative activities in facilitating the therapeutic process, with eight therapists perceiving creative activities as a valid, non-threatening and safe observation/assessment approach:

It takes the focus off while allowing me to ask questions and make observations. . .I consider whether their skills are developmentally appropriate. . . in self-directed activities with little/no perceived risk of failure. (Mary, S9)

The flexibility and fluidity of creative activities (*n* = 5) was valued by therapists, as was the ability for creative activities to be utilised as both a means of therapy and an ends (*n* = 6). Whilst not all therapists had a ‘specific goal in mind’, creative activities were identified as a powerful assessment and occupation-based medium where ‘the young person is free to express themselves without judgement, where their creativity is accepted’ (Vanessa, S21). This was further articulated by Ruth (S14) who viewed creative activities as a ‘relaxed, easy going, expressive activity, allowing for conscious or unconscious, hard to articulate feelings to surface and express outwardly’. An emphasis on the importance of theoretical frameworks to guide use of creative activities as a therapeutic medium was also identified (*n* = 3), in which occupation-based frameworks were identified:

A strong understanding of the application of occupationally-based frameworks as they apply to expressive media. . .allows the client to identify ways of moving forward, and space to experiment with new ways of thinking and being. (David, S6)

##### Barriers and enablers

Whilst therapists generally favoured the use of creative activities as a therapeutic medium, almost all (*n* = 30) identified workplace factors that either supported or hindered creative activity use. Particularly, funding and resource constraints (*n* = 21) including ‘limited facilities, resources, materials’ (Emily, S13), the impact of ‘time, preparation required’ (David, S6) and the ‘cost of materials and more funding’ (Bella, S18) were frequently identified. Therapists highlighted the influence of supportive organisational and environmental workplace dynamics and cultures on their use of creative activities. Sarah (S22) reported that ‘perceptions of team members and line management. . .and opportunities for professional development against pressure to use interventions with strong evidence as opposed to emerging’ supported creative activity use. Additionally, individual factors that either supported or hindered creative activity use including comfort with mediums was perceived to impact on therapists’ approach to creative activity use, as ‘Being confident with activities used – not using those I am not’ (Ruth, S14) was identified as an important consideration influencing creative activity use.

#### Legitimacy of creative activities within occupational therapy

##### Evidence-informed practice

Therapists voiced concern at the professions’ lack of identity and connection with creative activities, with many proposing that misconceptions of creative activities within occupational therapy, had contributed to the devaluation of creative activities as a legitimate therapeutic tool. Specifically, 9 therapists cited a lack of Australian occupational therapy studies, and 11 highlighted a lack of international discipline-specific evidence, education and training impacted creative activity use. Within this context, therapists highlighted ‘judgement by other professionals [of creative activities] as not being therapeutic’ (Bella, S18) and difficulties articulating and understanding ‘how to evaluate the effectiveness of creative therapies in CYMH’ (John, S15) were further barriers to using creative activities. Furthermore, a call for more discipline-specific evidence with ‘emphasis on using creative practices as occupation as means’ (Mary, S9) was suggested to promote exposure and understanding of creative activities in CYMH practice.

##### Ownership by the profession

An articulated sense of lost ownership of creative activities by the profession (*n* = 10) and a call for increased inter-professional and multidisciplinary collaboration and recognition of creative activities was conveyed (*n* = 13). One therapist commented:

A recent trend for interventions to be sensory focused, has decreased the focus creative activities once had within CYMHS. . .and other professions lack of valuing creative activity. . .and priority given to talking or cognitive therapies. The reduction in occupationally-based practice has resulted in a loss of framework. . .and a growth of other professions with ownership of creative therapies. (Ruth, S14)

#### Initial and ongoing professional development

##### Organisational/corporate governance

Access to formal and informal mentorship, supervision and training was considered pivotal to the skilful delivery, use and perceptions of creative activities (*n* = 15). At an organisational level, therapists reported that a ‘lack of resources, particularly lack of training and supervision’ (Olivia, S26) as well as ‘limited time to research various approaches. . .limited knowledge and opportunities and access to additional training’ (Steph, 25) impacted their ability to use creative activities in their practice. Therapists commented on the value of experiential learning through initial and ongoing professional development in supporting clinical knowledge (*n* = 13) and parent recognition of creative activities as a legitimate tool (*n* = 6), voiced by one therapist:

More professional development opportunities to upskill is needed, but it is expensive and not always recognised as core OT work. More opportunities for education of therapists and family members. . .awareness of the importance of creativity with young people and encouragement to use creative therapies is needed. (Tara, S16)

Additionally, Phoebe (S8) expressed ‘concerns that parents may not see [creative activities] as a viable therapeutic tool’. Therapists suggested that ‘opportunity for experiential learning in tutorials within the curriculum’ (David, S6) and provision of ‘training on practical skills and understanding of evidence-based practice’ (Louise, S2) would support students and clinicians.

##### Undergraduate education and training

Therapists (9) expressed a desire for greater education and training in creative activities at an undergraduate level. In reflecting on recommendations for training in creative activities for current and future students, one therapist reported: The only creative activity I recall doing at university was regarding children in therapy. Would perhaps be good for students to do a project where they are required to identify a way they could use creative activities with clients of different demographics (Mia, S3). A desire for knowledge translation articulated (*n* = 11) and further education/training needs (*n* = 8) was echoed by other participants who called for an emphasis on ‘practice applications and education materials based on recent evidence’ (Phoebe, S8) and the importance of ‘exposure to the possibilities [of creative activities]’ (Mary, S9).

## Discussion

This study examines Australian occupational therapists’ perceptions and use of creative activities in CYMH practice. The research findings revealed that therapists called for greater grounding in occupation-based frameworks to guide creative activity use; increased research examining the clinical utility and effectiveness of creative activities as an occupation-based intervention in CYMH; and more opportunities for education and training to enhance perceptions of creative activities and promote confidence and comfort in their use.

Therapists articulated a need for a strong grounding in occupation-based frameworks to guide the use of creative activities in CYMH practice. A felt sense of devaluation of occupation-based interventions from both within and outside the occupational therapy profession, at opposition with the professions core philosophy, was expressed by therapists to influence perceptions, comfort and confidence using creative activities. Several researchers have also expressed concerns regarding the marginalisation of occupational perspectives and occupation-based practices in favour of biomedical, psychological and perceived evidence-based practice modalities (Ashby et al., 2013; Copley et al., 2017; Kreider et al., 2014). Ashby et al. (2013) argue that the ‘dominant discourse’ of biomedical and psychological intervention methods, impact the professional resilience and identity of occupational therapists in mental health practice, which was reflected in the perceptions voiced by participants in this study.

Greater research specifically examining the efficacy of creative activity as an occupational therapy intervention for young people, is essential to reclaim occupation-based interventions at the centre of CYMH practice. A stronger grounding in occupation-based approaches could help less-experienced occupational therapists articulate their rationale for using creative activities and other occupation-based modalities in CYMH. Additionally, whilst there is a suggested resurgence of interest in occupation-based approaches in recent occupational therapy literature (Bathje, 2012; Copley et al., 2017), therapists in this study called for greater research into the efficacy of creative activities in CYMH practice.

Occupational therapists are expected to be guided by clinical experience, research evidence, client preferences and practice contexts in clinical decision-making to inform the use of specific practice interventions (Occupational Therapy Australia, 2019). Given the need for occupational therapists to implement evidence-based practice informed by theory, establishing the efficacy of creative activity use in CYMH occupational therapy is critical in helping therapists to conceptualise the way in which they use creative activities as a medium for transformational change. All therapists (*n* = 33) in this study considered creative activities to be highly efficacious in promoting positive mental health outcomes for children and youth and all reportedly used creative activities, compared to 44–82% reported internationally across practice settings in similar studies (Craik et al., 1998; Griffiths and Corr, 2007; Müllersdorf and Ivarsson, 2016). More research specifically examining the efficacy of creative activity as an occupational therapy intervention for young people through the direct measurement of therapy outcomes is essential. This research could help address challenges to role identity already articulated in other studies (Ashby et al., 2013; Hardaker et al., 2011), reduce clinical utility barriers and improve therapist confidence and comfort in creative activity use.

Finally, therapists in this study expressed a clear desire for greater education and training to enhance their confidence and comfort using creative activities. Emerging evidence supports the efficacy of arts in formative undergraduate and postgraduate occupational therapy education (Coppola et al., 2017). Such training has the potential to strengthen professional identity in the holistic use of creative activities in occupational therapy practice. In this study, statistically significant findings indicated higher levels of confidence and perceived expertise using creative activities were linked to provision of formal workplace training or undergraduate occupational therapy training. This is concerning, given that 84% of participants reported undertaking informal training to enhance knowledge and understanding of creative activities. Wimpenny et al. (2014) assert that inauthentic, un-examined and un-articulated practice can result in in-effective service provision and reduce health promotion and prevention benefits in mental health practice. Thus, greater access to formalised workplace training, mentorship/supervision and experiential learning opportunities to support confidence, comfort and skill levels in using creative activities as both a means and an end of therapy is needed. The influence of greater education and training, has the potential to improve the effectiveness of creative activity use in CYMH practice.

## Limitations

To our knowledge, this is the first study profiling Australian CYMH occupational therapists’ perceptions and use of creative activities. Whilst the study sought to engage CYMH occupational therapists in Australia nationally, therapists in Queensland or those who value creative activities may have been more likely to participate. Respondents in this study were predominantly public health CYMH workers, with approximately 19% of 100 available Queensland-based mental health special interest group CYMH occupational therapists having participated in this study. It is acknowledged that dissemination of the survey information by the Occupational Therapy Australia newsletter, may have missed valuable insights from non-members of the mental health special interest group and Occupational Therapy Australia network. Exclusion of therapists with higher education/degrees in music or art therapy may also limit applicability of findings to CYMH occupational therapy more broadly. Further, due to the small sample size involved, it is possible that alternate main categories may have been generated through a larger-scale study. Due to the nature of survey research and accessibility of membership data, prompting to achieve rich data and data saturation or estimates of response rates were not possible, identified as key limitations of this study. It is recommended that larger studies including surveys and interviews or focus groups be conducted, to further explore Australian and international occupational therapists’ perceptions and use of creative activities.

## Implications for practice and future research

In this study, Australian CYMH occupational therapists perceived creative activities to be a valuable and integral therapeutic medium. Whilst creative activities were highly utilised, therapists called for an increased evidence base, greater support for creative activity use as an occupation-based intervention and supervision/mentorship, training and education. To address these gaps, it is proposed that further survey and focus group research is conducted to explore the occupational therapy role, mechanisms of change, evaluation and implementation of creative activities in CYMH occupational therapy practice. There is a need for larger studies and more robust research to support consideration of inclusion of creative activities in the Australian occupational therapy curriculum to meet the professions demand for contemporary, occupation and activity-based training and evidence-informed modalities. The authors suggest embedding opportunities for experiential learning in creative activities and occupation-based frameworks and perspectives in CYMH workplace training and undergraduate occupational therapy education, to enhance therapists’ ability to deliver creative activities as an effective therapeutic medium. The study emphasises the value of selecting specific occupation-based and creative mediums to suit the practice context, child’s needs and provide opportunity for occupational growth. Overall, the study underscores the unique contribution of creative activities in occupational therapy as a powerful occupation-based therapeutic medium in CYMH practice.

**Key findings**

- Creative activities are perceived to be an efficacious therapeutic approach and are well utilised by Australian CYMH occupational therapists
- Training, mentorship and experiential learning could strengthen creative activity use

**What the study has added**

Fundamentally this study highlights the need to strengthen all elements of evidence-based practice to support the use of creative activities as a highly valued therapeutic medium in CYMH practice.

## Supplemental Material

sj-docx-1-bjo-10.1177_03080226251412760 – Supplemental material for Exploring Australian child and youth mental health occupational therapists’ perception and utilisation of creative activities: A survey snapshot Supplemental material, sj-docx-1-bjo-10.1177_03080226251412760 for Exploring Australian child and youth mental health occupational therapists’ perception and utilisation of creative activities: A survey snapshot by Hannah Forbes, Adam Lo, Michael Steele and Rosamund Harrington in British Journal of Occupational Therapy
