# Supplementary material for: Exploring Australian child and youth mental health occupational therapists’ perception and utilisation of creative activities: A survey snapshot
Source: Br J Occup Ther. 2026 Feb 26;89(11):728–37. doi: 10.1177/03080226251412760 (PMC13770165; doi:10.1177/03080226251412760)
Supplement: Supplementary file 1 [file sj-docx-1-bjo-10.1177_03080226251412760.docx]

**Appendices**

**Supplementary Material 1**

Australian Occupational Therapists Utilisation and Perception of Creative Activities Online Web-Based Survey Questions

| **Question Number** | **Question Details** | **Survey Response Options** | |
| --- | --- | --- | --- |
| **Q1** | I have read and understand the above consent form. I certify that I am 18 years old or older. By clicking the “Next” button to enter the survey, I indicate my willingness to voluntarily take part in this study. | Yes  No | |
| **Q2** | What is your gender? | Female  Male  Other  Prefer not to answer | |
| **Q3** | Which range best describes your current age? | < 21  21-30  31-40  41-50  51-60  61+ | |
| **Q4** | How many years have you practiced in mental health within occupational therapy? | < 1 year  < 2 years  < 3 years  < 4 years  < 5 years  < 6-7 years  < 8-9 years  < 10-11 years  > 11 years | |
| **Q5** | What level of qualification do you currently hold? | Bachelor’s degree  Post-graduate diploma  Master’s degree  Doctoral degree  Other | |
| **Q6** | Which statement best describes your current employment status? You may select more than one response | Full-time  Part-time  Casual  Not working (retired)  Prefer not to answer  Other | |
| **Q7** | What is your primary place of employment? | Private Hospital  Public Hospital  Community Service Provider  State Government  Private Practice  Federal Government  Non-government organisation  Other | |
| **Q8** | What is your primary practice setting? | Acute Hospital Unit  Residential Rehabilitation  Transitional Living Unit  Community  Other | |
| **Q9** | Which statement best describes your level of specialisation in mental health? You may choose more than one response | Entry grade occupational therapist (3 years or less)  Experienced occupational therapist (3 years or more)  Senior occupational therapist  Head occupational therapist  Professionally qualified art or music therapist  Other | |
| **Q10** | For the purpose of this study, creativity will be defined as originative or productive engagement in activity, in which creative activities are aptly defined as any purposeful activity involving active participation using imagination and creative skills (Cynkin & Robinson, 1990; Tubbs & Drake, 2007). Have you used creative activities in practice? | Yes  No | |
| **Q11** | What age are the clients for whom you use creative activities? You may select more than one response. | 0-4  5-8  9-12  13-15  16-18 | |
| **Q12** | How likely are you to use creative activities with children and youth with the following conditions? You may select multiple responses | Depression  Anxiety  Mood disorders  Attachment disorders  Social phobia  Obsessive compulsive disorder  Psychotic disorders  Other | |
| **Q13** | What area/s do your client's most commonly experience difficulties with? You may choose more than one | Self-esteem  Motivation  Concentration  Social skills  Self-management  Interpersonal skills  Other | |
| **Q14** | How often would you use creative activities as an intervention? | Less than once a month  At least once a month  At least once a fortnight  Multiple times a week  Daily  Multiple times daily | |
| **Q15** | Please rate how effective you perceive creative activities are in promoting positive mental health outcomes for children and youth that you work with. | 0 (least effective)  1  2  3  4  5 (moderately effective)  6  7  8  9  10 (most effective) | |
| **Q16** | Please rate how confident you feel in using creative activities in child and youth mental health occupational therapy practice. | 0 (very low level of confidence)  1  2  3  4  5 (moderate level of confidence)  6  7  8  9  10 (very high level of confidence) | |
| **Q17** | Please rate your level of expertise in delivering and using creative activities in child and youth mental health Occupational Therapy practice. | 0 (very low level of expertise)  1  2  3  4  5 (moderate level of expertise)  6  7  8  9  10 (very high level of expertise) | |
| **Q18** | What types of creative activities do/have you used? Please select all activities that you use/have used in practice and what category you feel these best fit | Painting  Artwork  Drawing/Colouring  Collage  Paper-craft  Printing  Photography  Calligraphy  Sewing  Patchwork  Knitting  Pottery  Sculpture  Drama  Creative Writing  Dance  Music  Other | Art-Based  Paper Based  Fabric Based  Dough Based  Word Based  Music Based  Not using activity  *(Activities were selected from the previous column and categorised into the above domains or practice activity type)* |
| **Q19** | What do you aim to achieve the most when using creative activities with children and youth? Please drag and drop the descriptors in order of most important aim (1) to least important aim (8) | Self-esteem  Self-confidence  Social engagement  Therapeutic relationship  Sense of achievement  Enhancing task performance  Self-expression  Assessment | |
| **Q20** | When using creative activities with your clients, what outcomes do you expect to achieve? | Open response question | |
| **Q21** | Do you use creative activities to the extent you would like to? | Yes  No | |
| **Q22** | What/if any barriers have you previously/currently experienced using creative activities with children and youth in Occupational Therapy practice in Australia? | Open response question | |
| **Q23** | What factors have previously/currently enabled your experience of using creative activities with children and youth in Occupational Therapy practice in Australia? | Open response question | |
| **Q24** | In your undergraduate degree/masters study to become an Occupational Therapists did you receive formal education/training on using creative activities? | Yes  No | |
| **Q25** | Have you received formal education/training (workshops, courses, degrees) on using creative activities in practice at your current workplace? | Yes  No | |
| **Q26** | Have you ever/are you undertaking informal education/training (journal articles, books, self-education) on using creative activities in practice? | Yes  No | |
| **Q27** | Have you pursued further training (workshops, courses, degrees) in the use of creative activities through another organisation? | Yes  No | |
| **Q28** | Would you feel more confident to use creative activities in practice if you were provided additional education/training? | Yes  No | |
| **Q29** | What if/any recommendations would you suggest for training in creative activities for current and future students undertaking an undergraduate bachelor's degree in Occupational Therapy? | Open response question | |
| **Q30** | What if/any recommendations would you suggest to improve/promote creative activities in Occupational Therapy practice in Australia? | Open response question | |
| **Q31** | Thankyou for your participation in this survey. If you would be willing to be contacted to participate in a follow-up individual semi-structured interview with the research team, please provide your preferred method of contact below. | Open response question | |

**Please note information regarding the Australian State/Territory that survey respondents resided in was recorded automatically by the Online survey platform Qualtrics. Participants were made aware of this in the consent form and participant information letter.*
